# Invasive Fungal Disease in Patients with Myeloid Malignancies: A Retrospective Cohort Study of a Diagnostic-Driven Care Pathway Withholding Mould-Active Prophylaxis

**DOI:** 10.3390/jof8090925

**Published:** 2022-08-31

**Authors:** Elizabeth A. De Kort, Jochem B. Buil, Steven Schalekamp, Cornelia Schaefer-Prokop, Paul E. Verweij, Nicolaas P. M. Schaap, Nicole M. A. Blijlevens, Walter J. F. M. Van der Velden

**Affiliations:** 1 Department of Haematology, Radboud University Medical Center, P.O. Box 9101, 6500 HB Nijmegen, The Netherlands; 2 Department of Medical Microbiology, Radboud University Medical Center, 6525 GA Nijmegen, The Netherlands; 3 Center of Expertise in Mycology Radboudumc/CWZ, 6525 GA Nijmegen, The Netherlands; 4 Department of Radiology, Radboud University Medical Center, 6525 GA Nijmegen, The Netherlands; 5 Department of Radiology, Meander Medisch Centrum, 3813 TZ Amersfoort, The Netherlands

**Keywords:** invasive fungal disease, diagnostic-driven management, antifungal prophylaxis, galactomannan, haematological malignancies, invasive aspergillosis

## Abstract

Objectives: Patients receiving remission induction therapy for acute myeloid leukaemia (AML) are at high risk of developing invasive fungal disease (IFD). Newer therapies with targeted antileukemic agents and the emergence of azole resistance pose a challenge to the strategy of primary antifungal prophylaxis. We report the experience of a diagnostic-driven care pathway (DCP) for the management of IFD in these patients, using only culture-directed mould inactive prophylaxis. Methods: Retrospectively, we used a single-centre study of consecutive patients receiving intensive chemotherapy for myeloid malignancies between 2014 and 2021. DCP consisted of serial cultures and serum galactomannan (sGM) screening, CT imaging, and bronchoscopy to direct targeted antifungal treatment. IFD was classified according to the 2020 EORTC/MSGERC criteria. Results: A total of 192 patients with myeloid malignancies received 300 courses of intensive chemotherapy. There were 14 cases of invasive yeast infections and 18 of probable/proven invasive mould disease (IMD). The incidence of probable/proven IMD during the first cycle of remission-induction chemotherapy was 4.6% (n = 9). sGM remained negative in all cases of invasive aspergillosis (IA), with positive mycology findings in bronchoalveolar lavage. All-cause mortality was 9.4% (n = 18) 100 days after starting chemotherapy and was comparable between patients with or without IFD. The fungal-related mortality was 1% (n = 2). Conclusion: Diagnostic-driven based management without universal mould active prophylaxis is a feasible strategy in the management of IFD and limits unnecessary antimould treatment during intensive chemotherapy. The poor performance of serial serum galactomannan screening in detecting IA warrants further investigation.

## 1. Introduction

Invasive fungal disease (IFD) is a common and potentially life-threatening complication in the haemato-oncology population. The incidence of IFD caused by moulds varies between 3% and 10% in patients receiving remission induction therapy for acute myeloid leukaemia (AML) or myelodysplastic syndrome (MDS), with a mortality rate reaching 30% in some studies [1,2,3]. Despite epidemiological shifts following the introduction of prophylaxis, *Aspergillus* remains the most common causative organism for mould infections and *Candida* for fungal infections caused by yeasts [4]. Primary prophylaxis with a mould-active azole drug has proven effective in reducing the incidence of IFD altogether and is recommended by numerous guidelines in haematological patients at the highest risk of developing IFD [5,6]. Still, the emergence of azole resistance and breakthrough fungal infections challenge the universal application of azole prophylaxis. More importantly, azole drugs have significant drug–drug interactions with various targeted antileukemic therapies, raising concerns about added toxicity and decreased effectiveness of antineoplastic treatments [7].

Diagnostic-driven care pathways (DCPs) directed towards *Aspergillus* detection and using mould-*in*active yeast prophylaxis, biomarker screening, and early CT imaging have been demonstrated to decrease the use of antifungal therapy without compromising survival [8]. They are, therefore, a promising alternative to universal mould-active prophylaxis. Moreover, while fever is the most common reason for starting empirical antifungal treatment, it is not always present. DCPs have also proven to be useful in the detection of IFD before or in the absence of fever, thus contributing to the early treatment of asymptomatic IFD [9,10]. Nevertheless, real-life data on the performance of DCPs are scarce as DCPs using biomarkers may not be applicable in every clinical setting, specifically in populations where a significant delay is expected in diagnostic testing results or where the prevalence of invasive aspergillosis (IA) is high and universal mould prophylaxis is indicated [11,12]. In our tertiary care institution, a DCP for the early detection of IFD was implemented almost a decade ago in patients receiving intensive remission induction therapy for high-grade myeloid malignancies. Importantly, primary antifungal prophylaxis is directed only at yeasts. In this retrospective analysis, we set out to describe the experience of this DCP consisting of *Candida*-gut-colonization-guided yeast prophylaxis, serial serum galactomannan (sGM) screening, and early diagnostic computed tomography (CT) imaging. The primary objective was the incidence of invasive mould disease (IMD), whilst secondary objectives included survival outcomes as well as the incidence of invasive yeast infections.

## 2. Methods

### 2.1. Patients and Treatment Protocol

We performed a retrospective analysis involving consecutive adult patients receiving remission-induction chemotherapy for myeloid neoplasia (MN), mostly AML, in our tertiary referral centre between January 2014 and February 2021. Data were collected both prospectively in institutional databases and retrospectively from electronic health records. Approval for this study was obtained from the local ethics committee, registration number 2013/064.

Remission-induction chemotherapy consisted of either standard-dose cytarabine combined with an anthracycline (idarubicin or daunorubicin; ‘3+7’-schema, HOVON study protocols [13,14], high-dose cytarabine with mitoxantrone (HAM), or intermediate-dose cytarabine (IDAC). Patients treated in national research protocols or not in complete remission after the first cycle (cycle I) received a second cycle (cycle II) of chemotherapy. A central venous catheter was used for intravascular access in all patients.

### 2.2. Diagnostic-Driven Care Pathway

**Figure 1:**
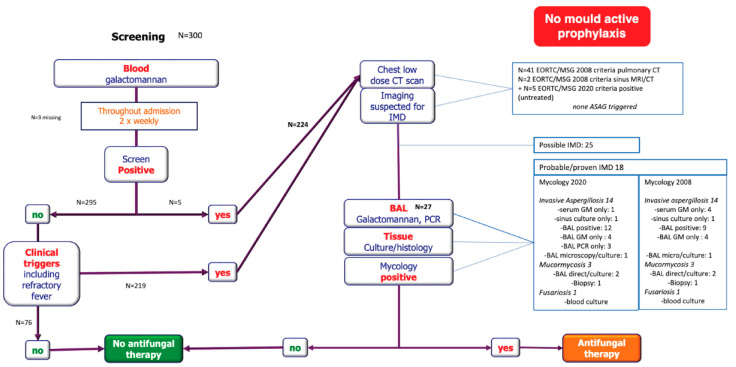
Radboudumc diagnostic-driven care pathway with stepwise overview of diagnostic testing and results. Positive diagnostic tests according to EORTC/MSG 2020 criteria except if stated otherwise.

All patients were managed with this standardized DCP from admission until neutrophil recovery. Antimicrobial prophylaxis directed at Gram-negative bacteria was given to all patients. In those colonized with *Candida albicans, C. tropicalis*, or *C. parapsilosis,* fluconazole (200 mg OD) was prescribed, whereas echinocandins were given in those colonized with fluconazole-resistant *Candida* species [15]. Mould-active prophylaxis was not routinely given during the cytotoxic-or neutropenic phases. sGM (Platelia assay), surveillance blood cultures, faecal swabs, and mouthwashes for bacteria and *Candida* species were collected and analysed twice weekly. Diagnostic low-dose chest CT was performed in patients with positive sGM (≥0.5), in those with recurrent or persisting fever despite 3–5 days of broad-spectrum antibacterial therapy, and in patients with progressive respiratory failure. If CT confirmed the presence of pulmonary infiltrates suspected for IFD based on standard radiology reporting, bronchoscopy and bronchoalveolar lavage (BAL) were performed if clinically feasible. Standard microbiology investigations included direct microscopy using fluorescent whitening staining, GM measurement, and fungal culture followed by susceptibility testing using EUCAST methodology if positive. In addition, from 2017 onward, PCR-based resistance detection on direct BAL material was implemented to rapidly detect azole resistance in *A. fumigatus*. Antifungal treatment was started while awaiting mycology results and was stopped if mycology remained negative (Figure 1). Voriconazole was the first-choice treatment option for patients with suspected IA and high-dose liposomal amphotericin B for mucormycosis. Treatment duration was at the treating physician’s discretion.

Detailed information about this DCP is given in the web-only Figure 1.

### 2.3. Classification

IFD was diagnosed, classified, and documented as either possible, probable, or proven according to the 2008 European Organization for Research and Treatment of Cancer/Mycology Study Group Education and Research Consortium (EORTC/MSGERC) criteria during the study period [16]. Cases were re-classified according to the 2020 definition for this retrospective analysis by two investigators, with disputes being resolved by consensus [17].

### 2.4. Outcome

Overall survival was determined at day 100 after the start of chemotherapy. Fungal-related mortality was defined as death due to IFD without other comorbid conditions believed to have contributed to death, as was described previously by Wingard et al. [18].

### 2.5. Statistical Analysis

Group comparisons were performed by chi-square or Fisher’s exact test as appropriate using SPSS (IBM, version 25, Armonk, NY, USA). Probability of overall survival was calculated using Kaplan–Meier estimates in R (R Foundation for Statistical Computing, version 3.6.2, Vienna, Austria) using log-rank test for comparison. *p*-values < 0.05 were considered significant.

## 3. Results

### 3.1. Patients and Treatment

**Figure 2:**
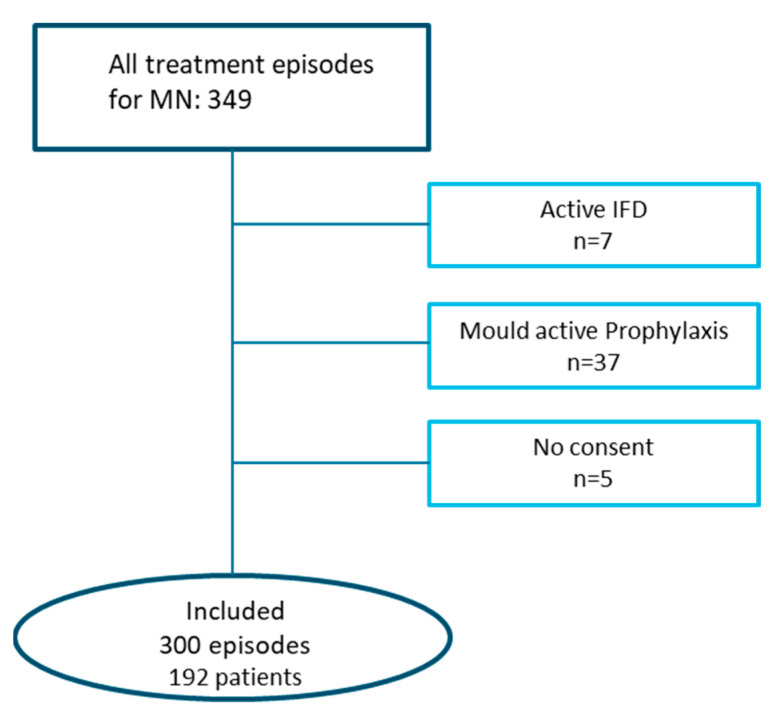
Study inclusion profile.

Overall, there were a total of 349 treatment episodes. After excluding patients lacking informed consent or those with a recent history of IFD requiring secondary prophylaxis and/or active IFD, 300 episodes or courses of treatment in 192 patients were included in this analysis (see web-only Figure 2). Candida-directed primary prophylaxis was given in 107 episodes (36%).

The characteristics of the patients and their treatments are summarized in Table 1.

### 3.2. Mould Infections

#### 3.2.1. Classification

Therapy was started for 43 mould infections. According to the 2008 EORTC/MSGERC definitions, there were 18 probable/proven cases and 25 cases of possible pulmonary mould infection (Table 2). The overall incidence of probable/proven cases was 9.3% (18/192 patients), with an incidence of 4.6% during cycle I (9/196 episodes) and 8.7% during cycle II (9/104 episodes).

Aspergillus was the most frequently detected pathogen (n = 14). No cases of azole-resistant IA were detected in patients with *A. fumigatus* infection.

When we applied the EORTC/MSGERC 2020 criteria, six cases were recategorized (four in cycle I, two in cycle II), but the number of cases per category remained unchanged. There were still 18 cases of probable/proven mould infections versus 25 cases of possible infections, with a similar distribution in chemotherapy cycles I and II (Figure 1).

There were three suspected cases where IMD could not be classified due to insufficient imaging criteria.

#### 3.2.2. Radiology

Chest CTs were performed in 224 cycles corresponding to 153 patients. They were predominantly performed because of persistent fever (93%, 208/224). Forty-one revealed pulmonary lesions suspected for IFD according to 2008 EORTC/MSGERC criteria. Two patients additionally had MRI features suggestive of fungal rhinosinusitis (one mucormycosis and one IA).

In retrospect, five patients had CT features (consolidations) that would have qualified them as having possible pulmonary mould disease according to the 2020 criteria. However, their condition improved without having received mould-active treatment and they were therefore categorized as not having IFD.

#### 3.2.3. Microbiology

Screening sGM was positive in 15 patients: median ODI 0.8 (range 0.5–6.1). In 10 patients, no clinical evidence for IMD could be found upon imaging (n = 8), autopsy (n = 1), or re-testing sGM(n = 1). In the remaining five patients, positivity was subsequent to CT imaging triggered by fever in all cases. When applying 2020 GM threshold values, only one of these results is considered positive.

Bronchoscopy with BAL was performed in 27 patients with pulmonary lesions suggestive of IFD and 6 patients with non-specific lesions. In the latter, BAL test results were negative for moulds. In the 27 patients with characteristic lesions, 9 had a GM-index ≥1.0, 3 had a positive culture for mould (1 *A. fumigatus*, 1. *A. flavus*, 1 *Rhizomucor pussilus*), and 1 had a positive PCR for *Rhizomucor*, Figure 1. In addition, eight patients had a positive PCR for *Aspergillus*, mostly *A. fumigatus*. The PCR result contributed to species identification in five patients with probable IA, and it was the sole positive test in three patients meeting the 2020 microbiological criterium for probable IA. sGM was negative in all but one case of IA diagnosed by BAL when applying the threshold of ≥0.5. When applying the 2020 criteria, sGM was negative in all cases. Only one of the 27 patients with suspected lesions was receiving prophylaxis with an echinocandin at the time of the BAL.

### 3.3. Yeast Infections

There were 5 proven, 1 probable and 8 possible invasive yeast infections; the characteristics of the 14 infections are summarized in Table 2 and detailed in Table 3.

The incidence of systemic *Candida* infections over the whole study period was 6.8% (13/192 patients); most infections occurred during the first cycle (cycle I, 6.1% (12/196 episodes) versus cycle II (1%; 1/104 episodes). During the study period, a seemingly high number of hepatosplenic candidiasis cases were observed, prompting the initiation of standard fluconazole prophylaxis at a dose of 400 mg OD starting the day after chemotherapy completion in December 2018. The incidence of systemic candidiasis dropped from 10% (13/131) before the introduction of standard prophylaxis to 0 cases in 61 patients treated thereafter (0%; Fisher test *p* = 0.01).

### 3.4. Outcome

After one hundred days after starting chemotherapy, 18 patients had died (9.4%). Only two deaths were considered fungal-related (probable IPA), leaving attributable mortality in the total cohort of 1%. There was no mortality attributable to systemic *Candida* infections.

Fungal-related mortality was 3.5% (2/57) in the IFD cohort and 11.1% (2/18) in those with probable/proven IMD.

**Figure 3:**
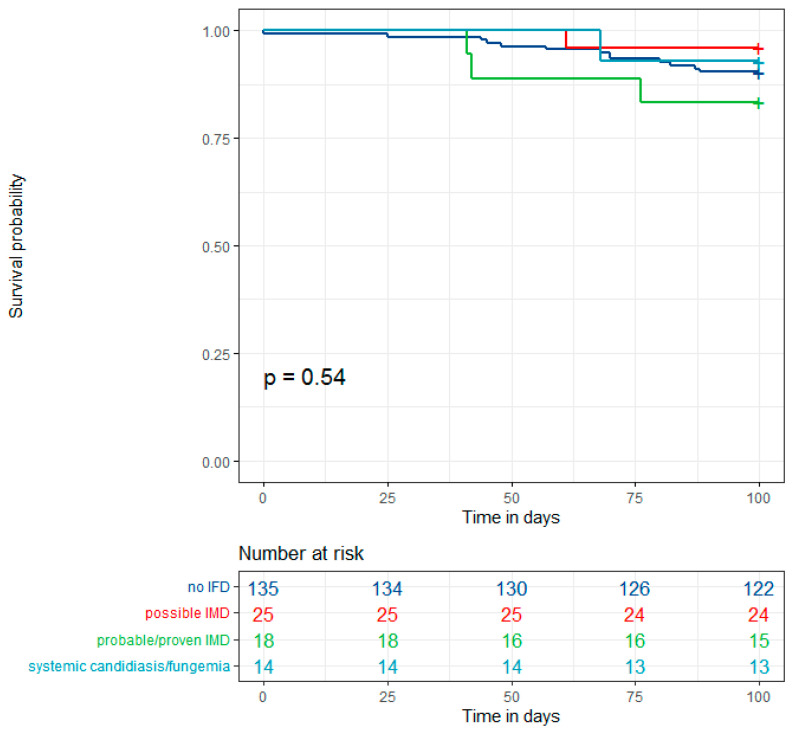
Kaplan–Meier 100-day survival curves according to IFD 2020 category.

The 100-day overall survival did not differ significantly in those experiencing IFD (*p* = 0.54) (Figure 3).

## 4. Discussion

Most recent studies have reported the epidemiology of IFD in patients receiving mould-active prophylaxis, but data on the performance of DCP are scarce. Herein, we present the incidence and outcome of a care pathway for IFD in a homogenous haematological population treated with intensive “3+7” chemotherapy between 2014 and 2021 and receiving only candida-directed prophylaxis.

Our diagnostic and care pathway using serial blood cultures, screening sGM, and clinical triggers for performing CT imaging reduced the use of mould-active agents to just 14% of cases undergoing intensive chemotherapy in a population which would have otherwise universally received prophylaxis with a mould-active agent. The rate of probable/proven IFD (including yeasts) in our population was 10.6 per 100 episodes of chemotherapy, which is comparable to that reported for patients with acute leukaemia [1,19]. While we failed to detect IFD before fever, early mortality did not differ significantly between patients experiencing IFD and those without, alluding to the safety of this DCP. Although the incidence of probable/proven mould infections of 4.6% in cycle I might have been too low to detect a significant difference in mortality, it is also far below the threshold of 8% at which mould-active prophylaxis is recommended by current guidelines, supporting our approach for early diagnosis and targeted therapy for IFD [5]. Still, there were some important caveats to this approach which led to a subsequent revision of our DCP.

**Figure 4:**
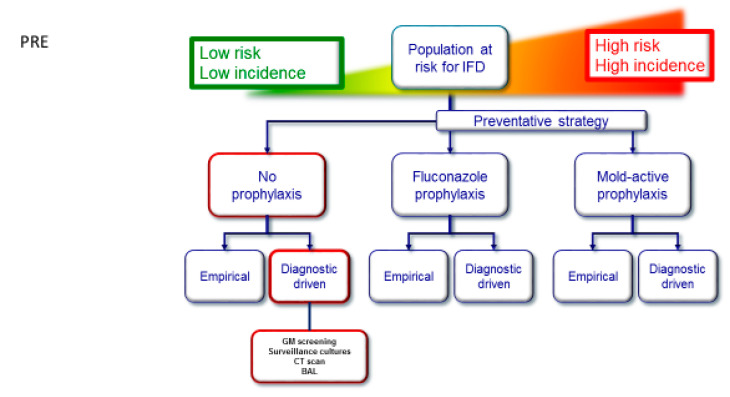

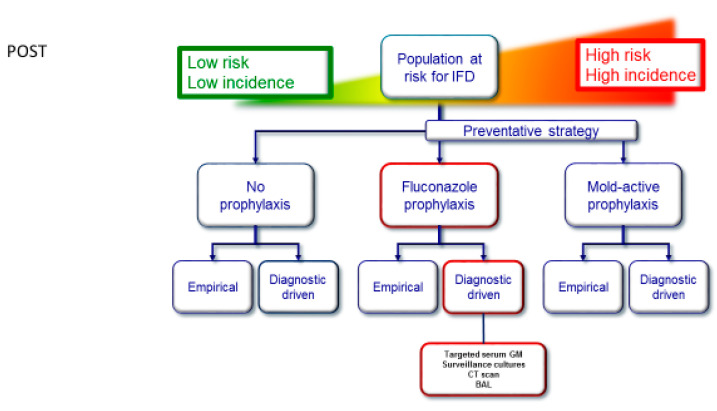
Radboudumc diagnostic driven pathway with a lower at-risk population for IA; pathway follows red tiles. Pre: before this analysis, using no standard antifungal prophylaxis. Post: after this analysis, alternative using standard fluconazole prophylaxis and targeted serum GM only to confirm suspected IA. This figure was adapted with permission from Agrawal et al. [8].

First, we observed a higher-than-expected rate of disseminated candidiasis compared to our own historical population (data unpublished). This was probably due to early fungal translocation from the gut before results of confirmation cultures were known and prophylaxis was started. After moving the start of yeast prophylaxis to earlier in the chemotherapy cycle, no more cases of invasive candidiasis were observed. Second, re-assigning cases according to the 2020 EORTC/MSG criteria established for research purposes redistributed IFD cases without affecting the total numbers in different categories of IFD. Due to the retrospective nature of this study, we can only speculate that this shift might have had an impact on the initiation or duration of antifungal treatment, and clinicians should be reluctant to downgrade treatment based solely on these criteria. Lastly, and most importantly, in contrast with early experience by Maertens et al., serial sGM monitoring was not effective in detecting IA in the absence of fever or clinical signs in our population [9]. All our cases were detected by CT imaging triggered mostly by persistent fever. Overall, sGM performed poorly for confirming IA, not able to detect IA in any of 12 patients diagnosed by BAL according to 2020 EORTC/MSG criteria, and we can only speculate on potential causes for this poor performance. An explanation might lie in the low pre-test IA probability in our population, especially in the first treatment cycle, where the incidence of IA was 4.6% instead of the 13.9% reported by Maertens et al. Although there have been reports of ineffective sGM screening when using echinocandin prophylaxis, the proportion of patients receiving echinocandins in our population was too small to have contributed to the poor performance [20]. Additionally, routine CT imaging for persistent fever might have led to earlier BAL before fungal burden could be detected by sGM. Indeed, sGM positivity followed suspicious CT findings in 5 out of 41 patients, suggesting an advantage of contemporary CT imaging for the early detection of IA (i.e., before angio-invasion) over serum biomarkers. In fact, CT imaging as a screening method of IA is being studied by different groups, and unlike sGM, it is non-invasive, vastly available, and offers quick results [21]. Despite the fact that newer assays for sGM, such as sandwich chemiluminescent immunoassays or lateral flow devices, offer faster turnaround times at comparable performance, based on our results, they are unlikely to contribute as a screening method. Nevertheless, these tests might provide mycological evidence in patients not able to undergo a bronchoscopy or additional tissue sampling. We therefore propose a new DCP screening method relying mostly on CT imaging as an early evaluation of neutropenic fever instead of screening sGM and apply the latter only as a targeted diagnostic test in patients with suspected CT findings (DCP, see web Figure 4).

The biggest advantage of our approach is that it can be tailored to various haematological populations (i.e., new hosts) and treatments. By withholding active mould prophylaxis, we successfully reduced the exposure of our haematology population likely to receive FLT3-inhibitors, IDH-inhibitors, or other antineoplastic drugs to potential drug interactions with azoles. While we did not detect azole resistance in our population, there are increasing numbers of azole-resistant *Aspergillus* strains detected in the Netherlands, Belgium, and the United Kingdom [22,23]. This pathway includes an aggressive approach to swiftly diagnose and manage (breakthrough) fungal infections and incorporates routine azole resistance testing, as described in the Figure 1.

In conclusion, the ECIL recommends active mould prophylaxis in patients at the highest risk for IFD, i.e., AML patients or the population with a risk ≥ 8%. We have shown that in our highest-risk population, the IFD burden can be considerably lower, and it is feasible to limit the exposure to azole drugs by adhering to a comprehensive diagnostic care pathway instead of giving universal mould-active prophylaxis.

## Figures and Tables

**Table 1:** Patient and treatment characteristics.

Characteristic	192 Patients
Patient age, median (IQR), y	57 (46–63)
Male sex, no (%)	110 (57%)
**Diagnoses, no. (%)** - AML - MDS-EB - Other o CMML-2 o Atypical CML o Histiocytic sarcoma o AUL/MPAL o MPN-BP/CML-BP	157 (82%) 18 (9%) 17 (9%) 2 2 2 6 5
**Treatment courses, ** **no.** - RI cycle I: o De novo MN o Relapsed AML - RI cycle II	196 193 3 104
**Treatment year, no.** - 2014–2015 - 2016–2017 - 2018–2019 - 2020–2021	53 55 59 25
***Candida−*** **directed prophylaxis,** **no. (%)** - fluconazole - echinocandin	107 (36%) 93 14
**Mould directed prophylaxis**	0

**Table 2:** Invasive fungal disease (EORTC/MSGERC 2008 and 2020).

Mould Infections	
Median time to diagnosis (days)	20 (IQR 17–26)
Possible IMD	16
Probable IMD o Invasive aspergillosis o pulmonary aspergillosis o rhinosinusitis o invasive mucormycosis o Pulmonary *Rhizomucor* spp.	16 13 1 2
Proven IMD o Mucormycosis Co-infection *Lichtheimia spp* and *Aspergillus* flavus o Fusariosis	2 1 1
Probable/proven IMD, no (%) - Patient (N = 192) - RI cycle I (N = 196) - RI cycle II (N = 104)	18 (9.3%) 9 (4.6%); 7 IA (3.6%) 9 (8.7%); 7 IA (6.7%)
**Yeast infections**	
Median time to diagnosis in days	30 (IQR 16–43)
Fungaemia o Candidaemia (*C. albicans, C. dubliniensis*) o *Geotrichum capitatum*	4 (3,1) 1
Disseminated hepatosplenic candidiasis o Possible candidiasis o Probable candidiasis	8 1
Systemic *Candida* infection Patient (N = 192) o Before 12-2018 (N = 131) o From 12-2018 (N = 61) - RI cycle I (N = 196) - RI cycle II (N = 104)	13 (6.8%) 13 (10%) 0 12 (6%) 1 (1%)

**Table 3:** Characteristics of patients experiencing candidiasis.

Patient nr	Age (Years)	Gender	Diagnosis	RI Cycle	Candidiasis	EORTC/MSG 2020 Category
1	60	F	AML	I	Hepatosplenic candidiasis	Possible
2	66	M	AML	I	Hepatosplenic candidiasis	Possible
3	59	M	aCML	I	Candidaemia	Proven
4	50	M	MDS-EB2	I	Hepatosplenic candidiasis	Possible
5	27	F	AML	II	Candidaemia	Proven
6	54	M	AML	I	Hepatosplenic candidiasis	Possible
7	64	F	AML	I	Hepatosplenic candidiasis	Probable
8	56	M	AML	I	Hepatosplenic candidiasis	Possible
9	40	M	AML	I	Hepatosplenic candidiasis	Possible
10	42	F	AML	I	Hepatosplenic candidiasis	Possible
11	60	F	AML	I	Candidaemia	Proven
12	60	F	AML	I	Candidaemia	Proven
13	44	F	AML	I	Hepatosplenic candidiasis	Possible

RI remission induction. aCML atypical chronic myeloid leukaemia; MDS-EB myelodysplastic syndrome with excess blasts; AML acute myeloid leukaemia.

## Data Availability

Data will be available by reasonable request directed to the corresponding author.
